# The effect of barium titanate-coated titanium alloy scaffolds on bone regeneration in osteonecrosis of the femoral head models: a comprehensive analysis based on *in vitro* and *in vivo* experiments

**DOI:** 10.3389/fbioe.2025.1671695

**Published:** 2025-09-12

**Authors:** Yu Chen, Yangfei Ou, Chunxing Xian, Hao Wu, Guoqing Pei, Wei Li, Ling Wang, Lei Shi

**Affiliations:** ^1^ Department of Orthopaedics, Xijing Hospital, The Air Force Medical University, Xi’an, Shaanxi, China; ^2^ Department of Orthopaedics, Tangdu Hospital, The Air Force Medical University, Xi’an, Shaanxi, China; ^3^ Department of Spine, Hanzhong Central Hospital, Hanzhong, Shaanxi, China; ^4^ Department of Health Statistics, Faculty of Preventive Medicine, The Air Force Medical University, Xi’an, Shaanxi, China

**Keywords:** osteonecrosis of the femoral head (ONFH), titanium alloy scaffolds, barium titanate (BaTiO_3_), bone regeneration, piezoelectric properties, macrophages, osteoclast differentiation

## Abstract

Osteonecrosis of the femoral head (ONFH) is a common condition that greatly affects patients’ quality of life, yet current treatments often have limited effectiveness. This study aimed to explore how a porous titanium alloy scaffold coated with barium titanate (BaTiO_3_) could promote bone regeneration in ONFH. We employed various research methods including cell culture, piezoelectric property measurements, tissue-engineered scaffold fabrication, and *in vitro* and *in vivo* biocompatibility assessments. Our results showed that macrophages had better attachment and growth on the BaTiO_3_-coated porous titanium alloy (PTB) scaffold than on the uncoated porous titanium alloy (PT) scaffold, with no significant differences in apoptosis rates between the two groups. Furthermore, the PTB scaffolds reduced the expression of bone resorption markers, such as Cathepsin K, TRAP, and RANK, under dynamic loading conditions. This finding indicates their potential to inhibit osteoclast differentiation. Moreover, the BaTiO_3_ coating enhanced the mechanical properties and biocompatibility of the scaffolds, evidenced by significantly higher alkaline phosphatase activity and calcium nodule formation in MC3T3-E1 osteoblasts cultured on PTB scaffolds. These findings underscore the dual role of BaTiO_3_ in facilitating cellular responses and modulating signaling pathways involved in bone metabolism. Our study highlights the promise of BaTiO_3_-coated titanium alloy scaffolds as an innovative approach to enhance bone regeneration in ONFH, paving the way for future clinical applications and the development of advanced biomaterials for bone healing.

## 1 Introduction

This study addresses the critical issue of osteonecrosis of the femoral head (ONFH), particularly focusing on innovative treatment strategies aimed at enhancing bone regeneration using barium titanate (BaTiO_3_)-coated porous titanium alloy (PTB) scaffolds. The significance of addressing ONFH is underscored by its high prevalence and the detrimental impact it has on patients’ quality of life, often leading to debilitating complications, including chronic pain and the need for surgical interventions such as hip arthroplasty (Hamilton et al., 2009). Current therapeutic approaches have shown limited effectiveness, highlighting an urgent need for advanced biomaterials that can significantly improve outcomes in bone regeneration (Fink et al., 1997).

Recent advances in biomaterials science suggest that the electrical properties of materials can play a pivotal role in modulating cellular responses during the bone healing process (Khare et al., 2020). Traditional studies have explored various biomaterials for bone regeneration; however, the influence of piezoelectric characteristics on cellular behavior remains underexplored (Tandon et al., 2018). BaTiO_3_, known for its piezoelectric properties, presents a promising avenue for research, as its ability to generate electrical signals could enhance the growth and differentiation of osteogenic cells (Sood et al., 2023; Mao et al., 2022).

The primary objective of this research is to evaluate the effects of PTB scaffolds on the cellular dynamics involved in bone regeneration, specifically focusing on osteoblasts and macrophages’ responses. The study employs a multifaceted approach, incorporating cell culture, piezoelectric property assessment, scaffold fabrication, and biocompatibility evaluations both *in vitro* and *in vivo* (Zhou et al., 2023). By integrating material science with biological principles, this research aims to elucidate the mechanisms through which piezoelectric materials can influence bone regeneration processes.

The methodology encompasses the fabrication of PTB scaffolds, followed by rigorous testing of their mechanical strength, surface properties, and biological compatibility. The anticipated outcome is to establish that these scaffolds not only support cellular attachment and proliferation but also promote osteogenic differentiation and inhibit osteoclastic differentiation through enhanced signaling pathways (Srirussam et al., 2019; Boyce and Xing, 2008). This investigation seeks to provide foundational knowledge that could lead to the development of more effective treatment modalities for ONFH, ultimately improving patient outcomes and advancing the field of orthopedic tissue engineering (Sood et al., 2023).

In summary, this study aims to bridge the gap between materials science and regenerative medicine by exploring the potential of BaTiO_3_-coated scaffolds in enhancing bone regeneration in ONFH. The findings could pave the way for novel therapeutic approaches that leverage the unique properties of piezoelectric materials, addressing a significant clinical challenge in orthopedic practice (Tandon et al., 2018; Mao et al., 2022).

## 2 Materials and methods

### 2.1 Scaffold preparation

Porous titanium alloy (Ti6Al4V) (PT) scaffolds were fabricated using Selective Laser Melting (SLM). In brief, the structure of the alloy was designed using Computer-Aided Design (CAD) software. The scaffold for *in vitro* cell seeding had a diameter of 3.0 mm and a height of 4.0 mm. The rod for rabbit femoral head had a diameter of 3.0 mm and a height of 11.5 mm. The pore size was 800 μm, and the scaffold porosity was 75%. The CAD model was imported into the SLM system, and Ti6Al4V powder (particle size: 100 μm) was preheated in a 3D printer (BLT-S210). The scanning resolution was 40 μm, and the scaffold was printed layer by layer at a speed of 1,500 mm/s under an electron beam current of 200 μA. For the preparation of the BaTiO_3_ coating, a solution comprising 0.1 M barium hydroxide (Ba(OH)_2_) and 0.1 M potassium hydroxide (NaOH) was injected into a hydrothermal polytetrafluoroethylene autoclave, ensuring complete coverage of the PT scaffold. The autoclave was subsequently heated to 200 °C and allowed to cool to ambient temperature over a duration of 2 h. The PTB scaffold underwent polarization via the corona polarization technique, employing a polarization voltage of 11.5 kV for a period of 30 min.

### 2.2 Scaffold characterization

The PT scaffolds and the surfaces of BaTiO_3_ coatings were examined using Scanning Electron Microscopy (SEM, Quattro S, Thermoscientific, United States). Additionally, Energy Dispersive Spectroscopy (EDS) was utilized to analyze the elemental composition, including the types and spatial distribution of the elements present. Atomic Force Microscopy (AFM, Dimension Icon, Bruker, Germany) was used to characterize the surface roughness. X-ray Diffraction (XRD, Bruker D8 Advance A25, Germany) was employed for phase analysis of the surface and characterization of crystal grain size. A nanoindenter (G200, KLA, United States) was used to characterize the Young’s modulus curve. Micro-computed tomography (micro-CT, Skyscan 1276, Bruker, Germany) was used, and reconstruction was performed using CT-Nrecon software (Bruker, Germany). DataViewer 1.6.6 and CTAn 1.20.8.0 software (Bruker, Germany) were used to analyze the pore size and porosity of the scaffolds. A quasi-static d_33_ m (JKZC, China) was used to characterize the piezoelectric coefficient (d_33_).

### 2.3 *In Vitro* cellular response under piezoelectric conditions

#### 2.3.1 Cyclic loading of cell-scaffold complexes

**FIGURE 1:**
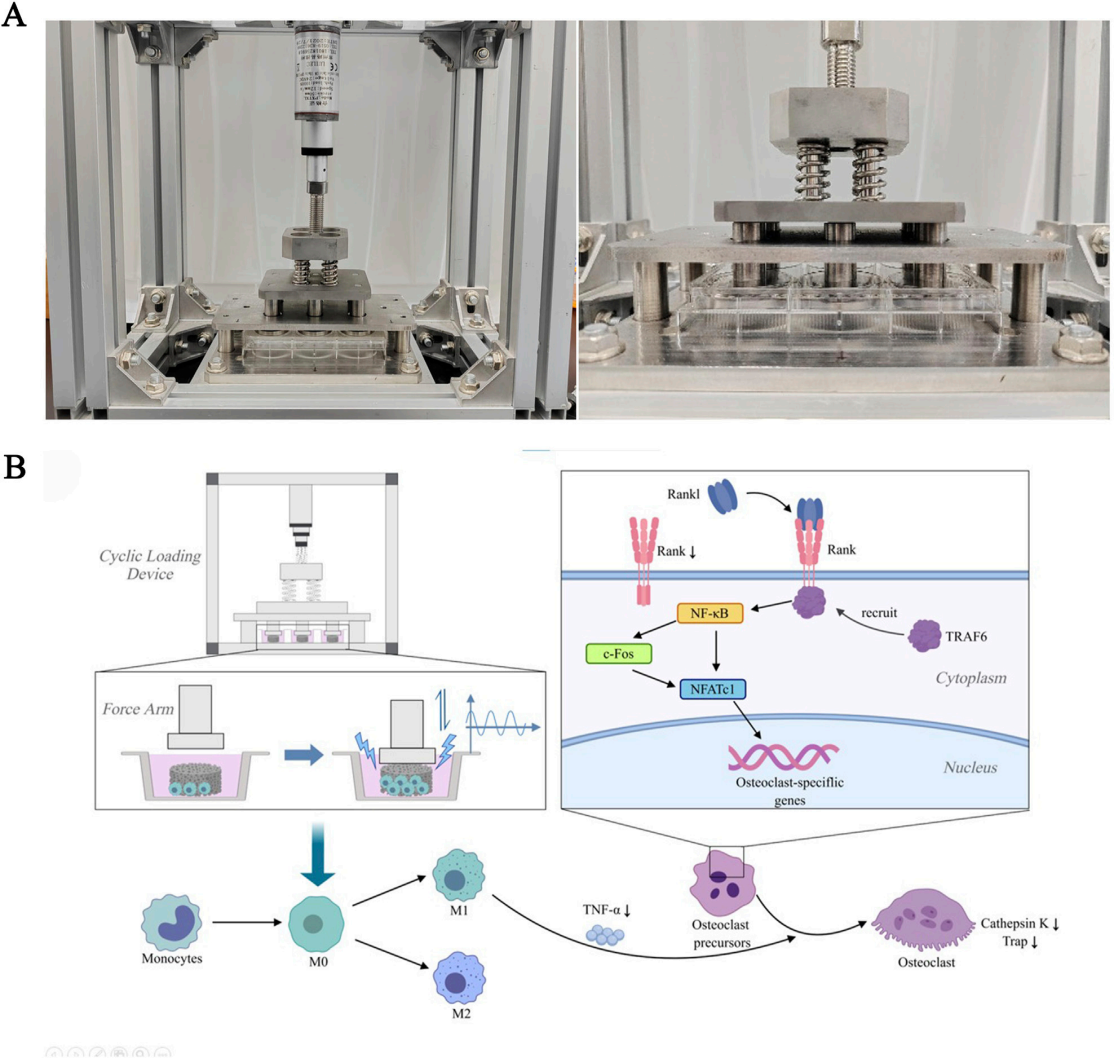
The RANKL/RANK signaling pathway in response to cyclic loading culture. **(A)** Cyclic loading device and the Force Arm. **(B)** Signal pathway diagram. The signaling pathways that contribute to osteoclastogenesis during piezoelectric stimulation activate NFATc1. RANKL binds to RANK on the surface of osteoclast precursors and recruits the adapter protein, TRAF6, leading to activation of NF-κB through phosphorylation and inactivation of inhibitory NF-κB inhibitory kinase, ultimately leading to the activation of c-Fos. NF-κB and c-Fos interact with the NFATc1 promoter, triggering the auto-amplification of NFATc1 and the transcription of genes, which mediate completion of the differentiation process. In addition to RANKL expression, macrophage/monocytes produce large amounts of TNF-α, which promotes osteoclast differentiation from osteoclast precursors. Piezoelectric stimulation can induce proliferation of macrophages and decrease the expression of cell surface receptor RANK and secretion of proinflammatory cytokine TNF-α, thereby inhibiting osteoclastogenesis.

To simulate the pressure environment of bone tissue *in vivo*, induce material deformation, and generate piezoelectric effects similar to natural bone, we designed a cyclic loading co-culture device. This device has been previously reported in an earlier article (Wu et al., 2023). A force arm controller regulates the pressure head to generate cyclic pressure, applying it to the material in the lower well plate, achieving a unidirectional stress effect similar to that in bone tissue *in vivo*. This causes slight deformation of the material, thereby achieving the piezoelectric conversion effect (Figure 1A). The loading parameters were selected as 1 Hz and 50 N (pressure intensity of 0.06 MPa).

#### 2.3.2 Cytotoxicity and biocompatibility

Cytotoxicity was determined by CCK8 assay. Dynamic extraction solution was added to macrophages (RAW264.7, iCell Bioscience Inc., China) suspension and co-cultured until 24,72,120 h. Absorbance (A) at 450 nm and relative cell proliferation rate (%) were monitored at these time points for all groups.

$$\text{Relative cell proliferation rate }\left(\%\right)=\frac{\text{Absorbance}\;\left(A\right)\;\text{of}\;\text{experimental}\;\text{group}}{\text{Absorbance}\;\left(A\right)\;\text{of}\;\text{negative}\;\text{group}}\times 100\%$$

Biocompatibility was evaluated by assessing the survival state of macrophages. Cells at a density of 1 × 10^5^/mL were seeded on PT and PTB scaffolds and co-cultured for 48 h, with daily 1-h stimulation using the cyclic loading device. Cells were stained using the Annexin V/PI staining kit (CA1020, Solarbio Science and Technology Co., Ltd., China), and apoptosis rates were detected using a flow cytometer (Attune™ Next, Thermo Fisher, United States) according to the manufacturer’s protocol. After staining with the calcein-AM/PI staining kit (CA1630, Solarbio Science and Technology Co., Ltd., China), cells were observed under a fluorescence microscope (Axioscope 5, Carl Zeiss AG, Germany), and images were processed using ZEN imaging software (Carl Zeiss, Germany).

#### 2.3.3 SEM observation

Macrophages at a density of 1 × 10^4^/mL were seeded onto the PT and PTB scaffolds. After incubation under dynamic loading (1 h/d) condition for 3 days, the macrophage-scaffold composites were fixed with 3% glutaraldehyde at 4 °C for 6 h, then dehydrated in a graded ethanol series, sputtered with gold and subjected to SEM observation. Macrophage were painted by pseudo color in pictures.

#### 2.3.4 Osteoclast induction and TRAP staining

Macrophages at a density of 1 × 10^5^/mL were seeded onto the PT and PTB scaffolds, with three replicates per group. Cells were incubated under dynamic loading conditions (1 h/day) for 3 days. Then the cells were detached and seeded into 6-well plates and placed in a cell culture incubator. 500 μL of complete culture medium containing RANKL (50 ng/mL) was added per well for induction. The medium was changed every 24 h, and after 5 days of induction, TRAP staining (G1492; Solarbio Science and Technology Co., Ltd., China) was performed according to the manufacturer’s protocol, and observed under a microscope for counting. Counting criteria: 1 Under a microscope at ×100 magnification, eight random fields were selected for counting without repetition. 2 Mature osteoclasts stained with TRAP appear rose-red or pink, with a nucleus count of ≥3.

#### 2.3.5 Western blotting

Proteins were extracted utilizing RIPA lysis buffer (BD0032; Bioworld Technology Co., Ltd., China), and their concentrations were determined with a BCA protein assay reagent (Top1003, Biotopped, China). An equal quantity of protein samples was subjected to separation through sodium dodecyl sulfate–polyacrylamide gel electrophoresis, followed by transfer onto nitrocellulose membranes. The PVDF membranes underwent a blocking procedure with 5% nonfat milk in Tris-buffered saline for 1 h, subsequently incubated overnight at 4 °C with primary antibodies targeting β-catenin (AF7018, Affinity, United States), Cathepsin K (DF6614, Affinity, United States), TRAP (DF6989, Affinity, United States), and RANK (DF12532, Affinity, United States). Following this, the membranes were treated with HRP-conjugated Goat Anti-Rabbit IgG (Proteintech, United States) for 1 h. β-actin (AF7018, Affinity, United States) served as the loading control. Detection of protein bands was achieved via chemiluminescence.

#### 2.3.6 Quantitative reverse transcription-polymerase chain reaction (RT-qPCR)

After the stimulation and induction period, the cells were collected and total RNA was extracted using RNAiso Plus (9108, Takara, Japan). The PrimeScript™ RT Master Mix (RR036A, Takara, Japan) was used for the reverse transcription reaction. TB Green^®^ Premix Ex Taq™ II (RR820A, Takara, Japan) was used to analyze the expression of CTSK (cathepsin K), ACP5 (TRAP), RANK. The expression levels of the target mRNA were normalized against those of GAPDH. The PCR conditions were as follows: 95 °C for 30 s, followed by 40 cycles at 95 °C for 5 s and 60 °C for 30 s. The primer sequences corresponding to each gene are detailed in Table 1.

**TABLE 1:** Primer sequences for RT-PCR

Gene		Primer sequence (5′-3′)	Length	Tm
CTSK	Forward	GTT​ACT​CCA​GTC​AAG​AAC​CAG​G	22	60.0
	Reverse	TCT​GCT​GCA​CGT​ATT​GGA​AGG	21	62.4
ACP5	Forward	GCA​ACA​TCC​CCT​GGT​ATG​TG	20	60.5
	Reverse	GCA​AAC​GGT​AGT​AAG​GGC​TG	20	60.7
RANK	Forward	CAT​CTT​CGG​CGT​TTA​CTA​CAG​G	22	60.7
	Reverse	TCC​ACT​TAG​ACT​ACT​GCA​AGC​A	22	60.8

#### 2.3.7 Osteogenic differentiation and ALP staining

The osteogenic precursor cells (MC3T3-E1, iCell Bioscience Inc., China) at a density of 1 × 10^4^/mL were seeded onto the PT and PTB scaffolds, with three replicates per group. Cells were incubated under dynamic loading conditions (1 h/day) for 7 days. The Alkaline Phosphatase Color Development Kit (P0321S, Beyotime, China) was used to perform alkaline phosphatase (ALP) staining. The ALP-positive cells were examined and photographed under a light microscope (Primostar 3, Carl Zeiss AG, Germany). An Alkaline Phosphatase Assay Kit (P0321S, Beyotime, China) was used to assess ALP activity. In brief, cells were lysed with Cell lysis buffer for Western and IP (P0013, Beyotime, China) on ice and centrifuged at 12,000 rpm and 4 °C for 15 min to collect the supernatant. A multifunctional microplate detector (Synergy H1, Biotek, United States) was used to measure the absorbance at 405 nm.

#### 2.3.8 Osteogenic differentiation and ARS staining

The MC3T3-E1 cells at a density of 1 × 10^4^/mL were seeded onto the PT and PTB scaffolds, with three replicates per group. Cells were incubated under dynamic loading conditions (1 h/day) for 7 days. After stimulation period, the cells were detached and seeded into 6-well plate, and cultured in a cell incubator for 7 days. Alizarin Red S (ARS) Staining Kit for Osteogenesis (C0148S, Beyotime, China) was used to detect the calcium deposits. The stained calcium nodules were observed under a light microscope. For quantitative analysis, 10% cetylpyridinium chloride (Aladdin) was used to solubilize the Ca-bound ARS for 1 h. The absorbance of the solution was measured at 562 nm using a spectrophotometer.

### 2.4 *In vivo* studies for assessing the therapeutic effect of the BaTiO_3_-coated porous Ti6Al4V rod against SONFH

#### 2.4.1 Rabbit model of SONFH

A total of 34 male New Zealand rabbits, with an average body weight of 3.5 kg, was utilized to develop the SONFH model. Briefly, all the rabbits were intravenously injected with Lipopolysacchanides (LPS, MedChemExpress, United States) (10 μg/kg). Methylprednisolone (MPS, MedChemExpress, United States) (20 mg/kg) was injected intramuscularly every 72 h. After MPS injection, cefazolin sodium (8 mg/kg) and Pantoprazole injection (10 mg/kg) are intramuscularly injected to prevent infection and peptic ulcers. The control group was administered an equivalent volume of normal saline. All drugs were injected 4 times. After 6 weeks of the start of the injection, all the rabbits were examined via micro-CT imaging. Four rabbits were then randomly selected and euthanized for histological assessment (H&E staining). All animal research was conducted with the ethical approval granted by the Ethics Committee of the Air Force Medical University (SYXK-2019-001).

#### 2.4.2 Implantation

**FIGURE 2:**
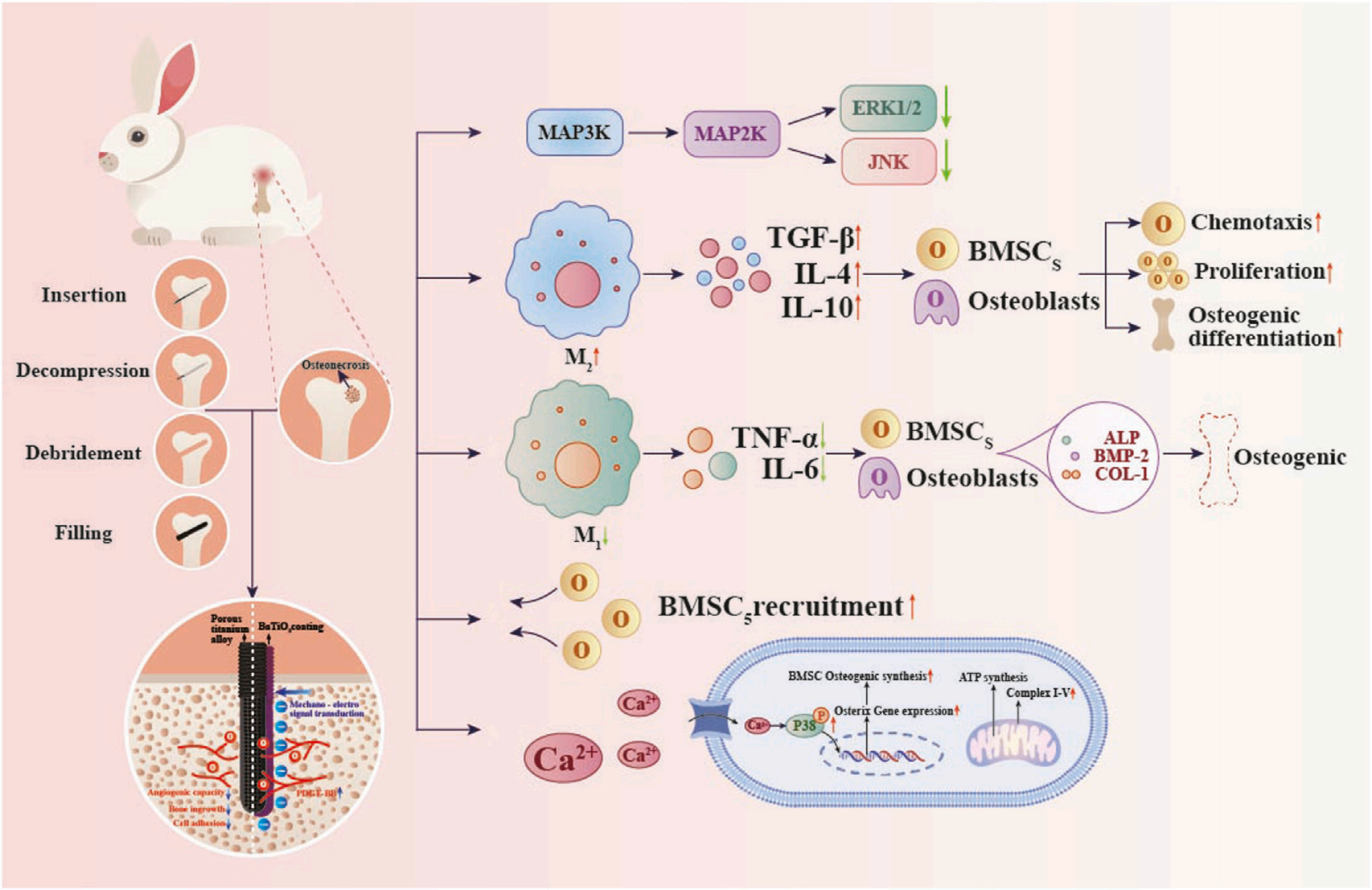
Molecular mechanisms by which a BaTiO_3_ piezoelectric coating on porous titanium alloy scaffolds modulates *in vivo* osteogenesis. Piezoelectric stimulation increases intracellular Ca^2+^ concentration and enhances phosphorylation of p38 MAPK, thereby upregulating Osterix expression and promoting osteogenesis. Increases PDGF-BB secretion, promoting bone angiogenesis. Inhibit the MAPK signaling cascade and enhance ATP production and oxidative phosphorylation to promote macrophage M2 polarization.

16 SONFH model rabbits were randomly divided into two groups. The PT group received Ti6Al4V rods, while the PTB group received BaTiO_3_-coated Ti6Al4V rods, with eight rabbits in each group. In brief, anesthesia was administered via intramuscular injection of Tylenol 100 at a dose of 0.1 mL/kg. Following skin preparation, disinfection, and draping, a longitudinal incision approximately 2.5 cm in length was made on the skin posterior to the greater trochanter of the femur. The superficial muscles attached to the posterior edge of the greater trochanter were carefully dissected, exposing the area from the posterior aspect of the greater trochanter to the femoral neck. The entry point was located 2 mm towards the femoral neck from the midpoint of the line connecting the greater and lesser trochanters. Using a 0.8 mm drill bit, a hole was drilled towards the femoral head at an angle of approximately 20° to the femoral surface, to a depth of about 12 mm. After drilling, the bit was removed, and a 0.8 mm positioning pin was inserted. X-ray fluoroscopy was performed in both anteroposterior and lateral views until the pin penetrated 1–2 mm into the subchondral bone of the femoral head. The hole was then sequentially enlarged using 1.5 mm and 3.0 mm hollow drill bits to create a cylindrical core decompression (CD) channel approximately 3 mm in diameter and 12 mm in depth. The rod was then tapped into the bone channel and rotated to secure them in place (Figure 2). The muscle, fascia, and skin were sutured in layers. Numbered tags were attached to the rabbits’ ears to distinguish between groups. Postoperatively, cefazolin sodium injection was administered intramuscularly at a dose of 25 mg per injection, once daily for three consecutive days.

#### 2.4.3 HE staining of the important organs

The organ tissue was put into 4% PFA fixed at weeks 12 after implantation, then embedded and sliced. The sections were dewaxed and placed in a high-definition staining pretreatment solution for 1 min. The sections were stained with hematoxylin solution for 3–5 min, then successively immersed in differentiation solution and bluing solution, followed by staining with eosin solution for 15 s. The sections underwent gradient dehydration and were mounted with neutral balsam. Observe and analyze the tissue morphology of organs, evaluate whether there is pathological damage of organs.

#### 2.4.4 Micro-CT analysis

Femoral head samples with rod were dissected and trimmed to appropriate size for micro-CT imaging. In brief, the samples were preserved in 4% polyformaldehyde prior to undergoing micro-CT. The scanning protocol was established with a resolution of 40 μm, a rotational angle of 180.0°, a voltage setting of 90 kV, and a current of 200 μA. Nrecon and CTAn software were used to 3D reconstruction and analyze the inner bone volume.

#### 2.4.5 Van gieson (VG) staining

Femoral head samples with rod were put into 4% PFA fixed for at least 48 h. The tissue is then dehydrated in 60%–70%–80%–90%–95%–100% alcohol and xylene. Put tissue into three cylinders of permeates penetrating fluid at 4 °C for 24 to 72 h in turn. The tissue was put into the embedding bottle, and embedding agent was added. Then vacuumed for 5 h and polymerized in a 37 °C water bath. Routine sectioning about 10 μm by hard tissue slicer (HistoCore AUTOCUT, Leica, Germany) and place sections at 60 °C oven overnight. Put sections into ethylene glycol ethyl ether acetate I for 6 h at 37 °C and ethylene ether acetate II overnight at 37 °C. Then put them into ethylene glycol ethyl ether acetate III for 10–15 min at room temperature and ethylene glycol ethyl ether acetate IV for 10–15 min at room temperature. The sections were rehydrated in 100%I%–100%II–95%–90%–80% alcohol. Finally, rinse with running water. Mixed with 9 mL of VG staining solution B and 1 mL of VG staining solution A. VG dyeing solution for 3 min, then rapid water washing, rapid dehydration with three tanks of anhydrous ethanol. Put in clean xylene transparent for 5 min, Finally, sealed the sections with neutral gum. Microscope observation and collection and analysis images.

### 2.5 Statistical analysis

Quantitative data are expressed as mean ± standard deviation (x ± s). Inter-group comparisons were analyzed using one-way analysis of variance (ANOVA), followed by the SNK-q test for pairwise comparisons. Rate comparisons were analyzed using the chi-square test. Statistical analysis was performed using SPSS 26.0 software, with P < 0.05 indicating statistical significance.

## 3 Results

### 3.1 Characterization analysis

**FIGURE 3:**
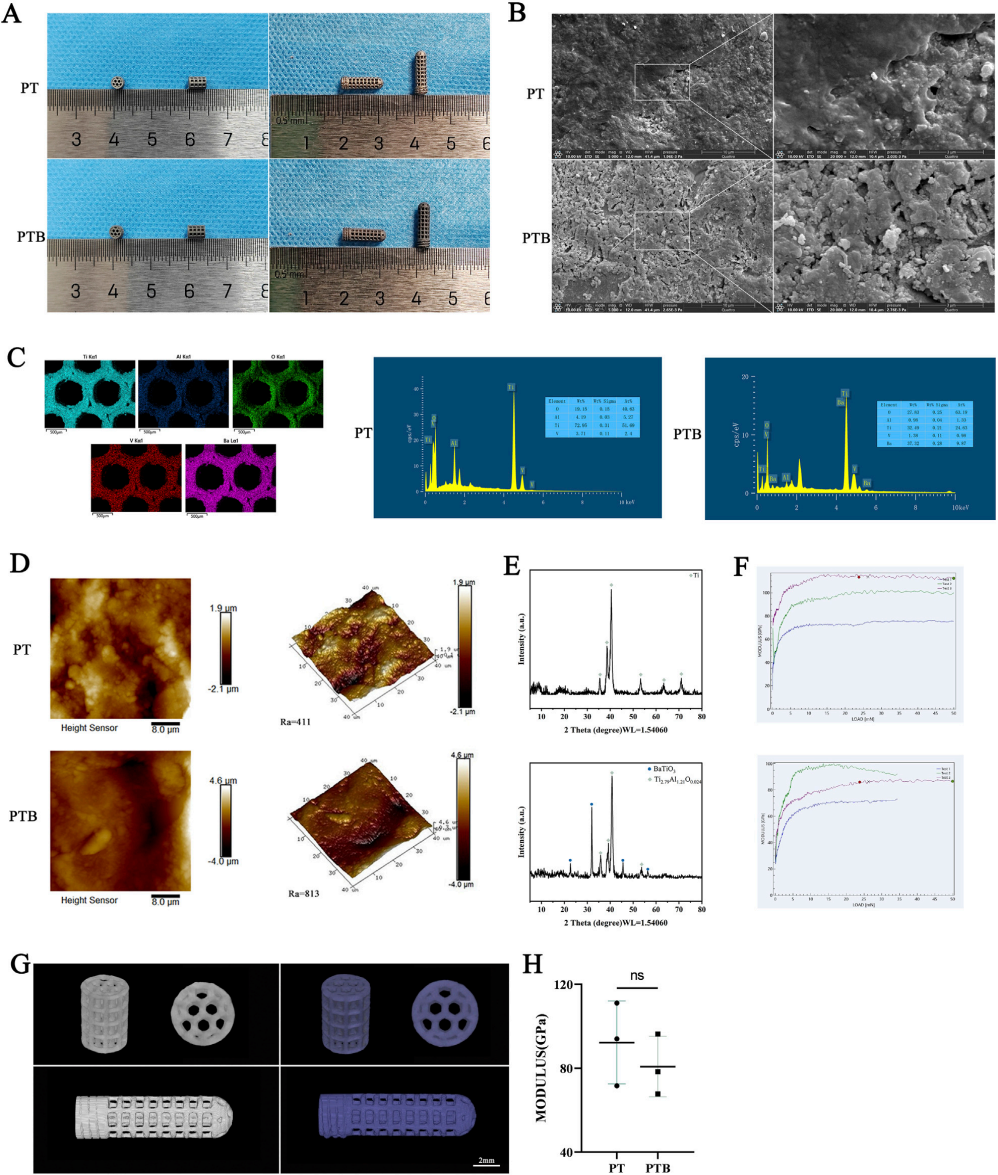
Scaffold Characterization. **(A)** General view of the 3D printed PT and PTB body scaffolds and rods. **(B)** SEM image of material surface (5,000x and 200,00x). **(C)** Distribution and Proportion of elements analyzed by EDS. **(D)** Surface roughness observed by AFM (2D and 3D images). **(E)** Detection of phase and crystal structure with XRD. **(F)** The Young’s modulus of the scaffold and statistical analysis. **(G)** Micro-CT 3D reconstruction image of scaffolds and rods. **(H)** The statistical diagram of the Young’s modulus.

The PTB scaffolds were dark blue (Figure 3A). The global structure of the PT scaffolds did not change considerably after BaTiO_3_ coating and polarization. BaTiO_3_ particles were evenly coated on the surface of the scaffolds, tightly integrating with the titanium alloy. The integration revealed granular and flake-like crystalline structures (Figure 3B). The regions where signals of Ti, Al, and V elements overlapped corresponded to the scaffold structure, while Ba elements confirmed the evenly presence of the surface coating (Figure 3C). The surface morphologies of the PTB group from two and three dimensions, indicating that the surface Roughness Average (Ra) of it increased (Figure 3D). The formation of typical 45° double peaks, indicating the tetragonal crystal phase of BaTiO_3_ (Figure 3E). The Young’s modulus was measured using a nanoindentation instrument, and the resulting curve showed no significant difference between the two groups (Figures 3F, H). Micro-CT results indicate that, after coating, the pore size and porosity of the PTB group slightly decreased, while the diameter of the metal fibers slightly increased. However, the differences in data between the two groups were not statistically significant. The uniform coating of BaTiO_3_ particles had little impact on the porous and strut structures of the titanium alloy (Figure 3G). The PTB group exhibited a piezoelectric constant of 0.667 ± 0.082, whereas no piezoelectric constant was observed in the PT group (Table 2).

**TABLE 2:** Main parameters (micro-CT) and piezoelectric constant (d_33_) of scaffold.

Group (n = 6)	PT	PTB	t	P
Pore Size (mm)	0.855 ± 0.087	0.844 ± 0.032	−0.277	>0.05
Wire Diameter (mm)	0.350 ± 0.033	0.356 ± 0.045	0.266	>0.05
Porosity (%)	74.548 ± 0.781	73.743 ± 0.420	−2.225	>0.05
d_33_ (pC/N)	-	0.67 ± 0.16	12.17	<0.0001

### 3.2 Cytotoxicity and biocompatibility of the scaffolds

#### 3.2.1 Cytotoxicity

**FIGURE 4:**
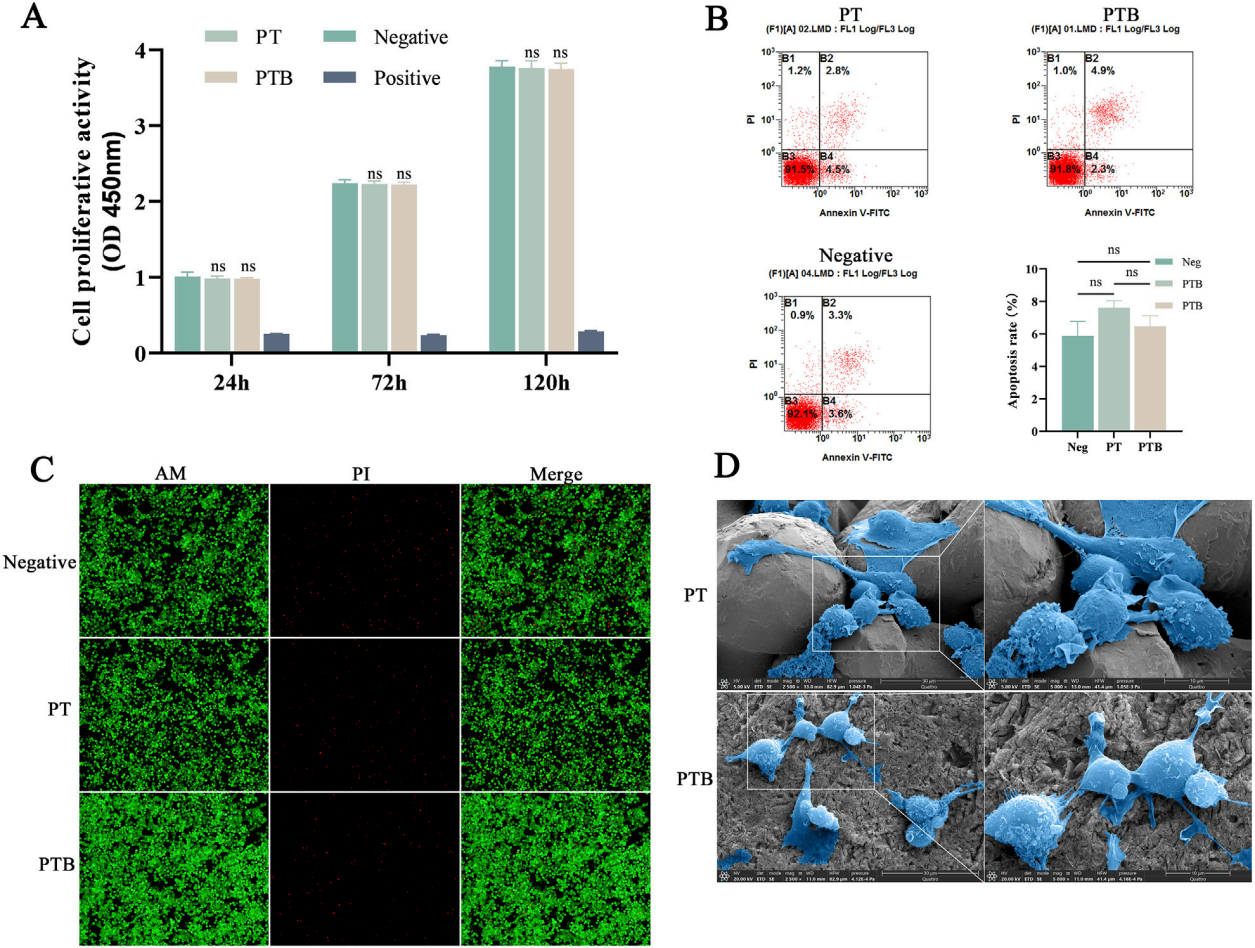
Scaffold biocompatibility. **(A)** The statistical diagram of CCK-8 for Absorbance Comparison at Each Time Point (n = 8). Ns, p > 0.05 vs. Negative group. **(B)** Flow Cytometry Analysis and statistical diagram of cell apoptosis rate in each group (n = 3). Ns, p > 0.05. **(C)** AM/PI Staining at ×100 magnifications. **(D)** SEM images of Macrophages grow on material surfaces (2,500x and 5,000x).

The absorbance of the PT, PTB and negative group all increased over time, with relative proliferation rates maintained at over 90%. In contrast, the absorbance values of the positive control group showed a downward trend over time. Comparisons of absorbance between the PT, PTB and negative group showed no significant differences (P > 0.05), suggesting that the extracts from the materials prepared by this process had no effect on *in vitro* cell proliferation (Figure 4A; Table 3).

**TABLE 3:** The absorbance (A) (x̅±x) and relative cell proliferation rate (%) (n = 8).

Group	24h		72h		120h	
	A	Rate (%)	A	Rate (%)	A	Rate (%)
PT group	0.986 ± 0.030	97.33	2.231 ± 0.043	99.52	3.761 ± 0.098	99.13
PTB group	0.982 ± 0.014	96.99	2.221 ± 0.034	99.07	3.747 ± 0.079	99.51
Negative group	1.013 ± 0.0564	100.00	2.241 ± 0.043	100.00	3.780 ± 0.080	100.00
Positive group	0.255 ± 0.003	26.53	0.240 ± 0.008	10.69	0.289 ± 0.011	7.63

#### 3.2.2 Biocompatibility

The PT group had an apoptosis rate of 8.63%, while the PTB group had a rate of 9.15%; neither was significantly different from the control group’s rate of 7.92% (P > 0.05). The differences compared to the control group (7.92%) were not statistically significant (P > 0.05) (Figure 4B). AM/PI Staining after Co-culture showed that, abundant green fluorescence was observed by both sets of results, with significantly higher intensity compared to red fluorescence. The PTB group exhibited higher intensity of green fluorescence than the PT group, while the intensity of red fluorescence was lower than that of the PT group (Figure 4C).

#### 3.2.3 Surface cell state by SEM

Cells exhibited robust growth and full morphology on both surfaces. Cells extending pseudopodia and adhering to the gaps between particles on the surface of the titanium alloy. Cells growing stably on the BaTiO_3_-coated surface, exhibiting flattened or elongated states (Figure 4D). The BaTiO_3_ nanoparticles coating on the titanium alloy surface provided attachment points for cell adhesion and growth.

### 3.3 Effects of scaffolds on cell repair *in vitro*

#### 3.3.1 Osteoclast induction by RANKL and TRAP staining

**FIGURE 5:**
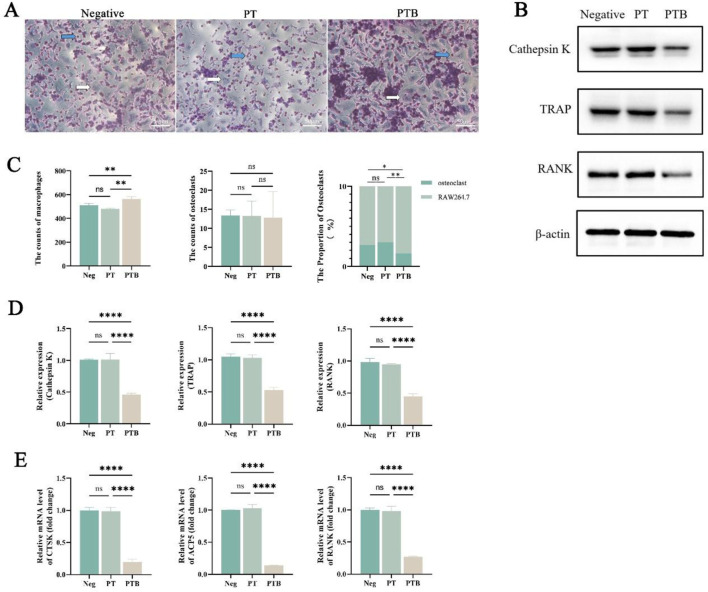
Osteoclastic induction. **(A)** Light microscopy images of TRAP staining after osteoclast induction (100x). The blue arrows in the figure indicate macrophages, while the white arrows represent osteoclasts. **(B)** Western blot analysis (n = 3). **(C)** Statistical graph of macrophage counts, osteoclast counts and the proportion of osteoclasts in the total cell count (n = 3). Ns, p > 0.05. *, p < 0.05. **, p < 0.01. **(D)** Statistical graph of the relative expression of Cathepsin K, TRAP and RANK (n = 3). Ns > 0.05, ****p < 0.0001. **(E)** Relative mRNA expression of osteoclast-related genes (CTSK and ACP5) and macrophage-related gene (RANK). Ns > 0.05, ***p < 0.001.

Numerous macrophages (blue arrow) were observed, with nuclei stained in purple-red, predominantly round or oval-shaped, and some displaying varied morphologies including pseudopodia. Large osteoclasts (white arrow), characterized by their blue-purple nuclei and light red cytoplasm, are scattered around. The cell count observed under the microscope shows that the number of osteoclasts is similar in both groups. However, statistical analysis reveals that the proportion of osteoclasts in the PTB group is lower than that in the PT group (Figures 5A,C).

#### 3.3.2 Piezoelectric scaffold activated the rank/rankl/OPG signaling pathway of osteoclast precursor under circulating load

To investigate the effect of BaTiO_3_ piezoelectric scaffolds on the osteoclast differentiation of macrophages *in vitro*, we measured the expression levels of osteoclast-specific proteins Cathepsin K, TRAP, and the receptor protein RANK in precursor osteoclast cells after co-culture and osteoclast induction (Figure 5B). Compared to the Neg group and PT group, the expression levels of CTSK, ACP5 and RANK in the PTB group decreased, while no statistically significant differences were observed between the Neg and PT groups (Figures 5D,E).

#### 3.3.3 ALP and ARS staining of osteoblast differentiation

**FIGURE 6:**
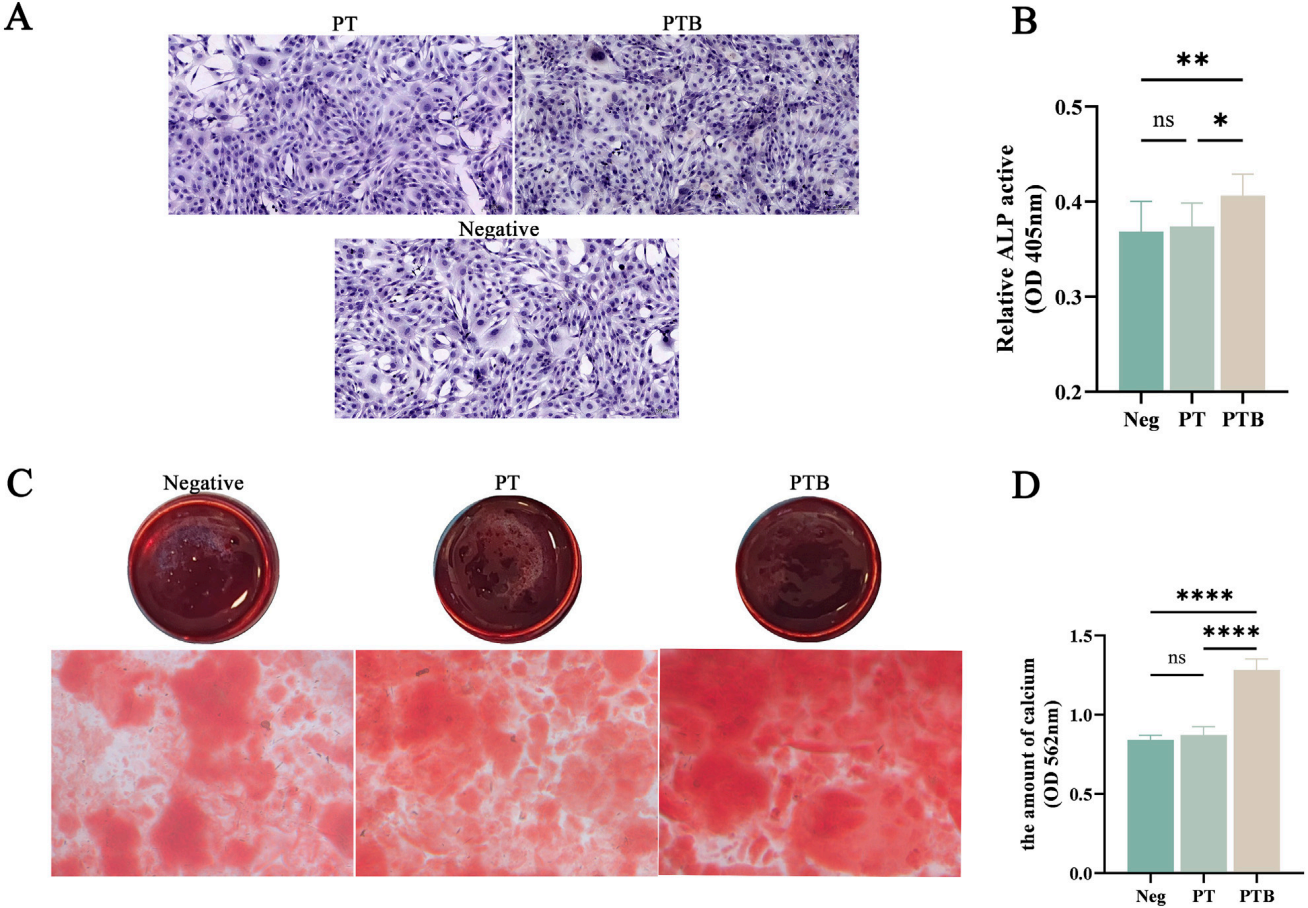
ALP and ARS staining of osteoblast differentiation. **(A)** ALP staining of Osteoblasts. **(B)** ALP activity analysis. **(C)** ARS staining of calcium **(D)** quantitative analysis of the calcium. Ns, p > 0.05. *, p < 0.05. **, p < 0.01. ****, p < 0.0001.

After 7 days of co-culturing MC3T3-E1 cells *in vitro*, the PTB group showed a significant increase in cell number and a much deeper ALP staining intensity compared to both the control and PT groups. The PTB group had higher ALP activity, while no statistically significant difference was found between the PT group and the negative control group (Figures 6A,B).

After 14 days of co-culturing MC3T3-E1 cells *in vitro*, ARS staining of calcium nodules showed extracellular matrix mineralization in all three groups. The PTB group exhibited a significantly greater formation of calcium nodules and deeper staining intensity. Quantitative analysis indicated that the calcium dissolution amount in the PTB group was higher than that in the blank group and the PT group. These results suggest that the piezoelectric stimulation from the PTB group can promote the proliferative activity of osteoblasts and the formation of calcium matrix (Figures 6C,D).

### 3.4 *In vivo* toxicity and bone regeneration of rods

#### 3.4.1 Evaluation of rabbit SONFH models

**FIGURE 7:**
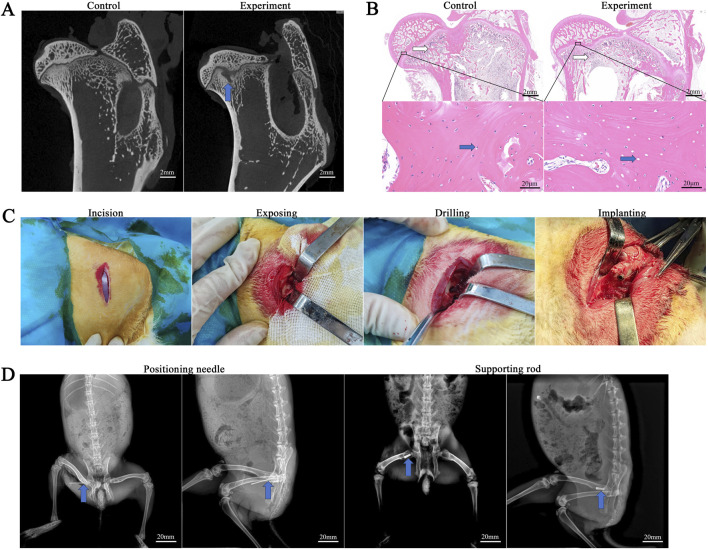
Evaluation of the rabbit SONFH model. **(A)** Micro-CT evaluation. The blue arrow shows necrotic area. **(B)** HE staining evaluation. White arrows indicate trabeculae, and blue arrows indicate lacunae. **(C)** The experimental procedure of rabbit rod implantation. **(D)** X-ray positioning during the experiment.

In the control group, the femoral head articular surface was clear, exhibiting smooth and intact cortical bone. The trabeculae were tightly interwoven, with osteocytes evenly distributed within their lacunae, and their nuclei were clearly visible. In the model group, the femoral head exhibited low-density areas within the trabecular bone and showed structural changes. In addition to low-density areas, some trabeculae were disorganized and loosely structured. Empty lacunae are present within the bone matrix due to osteocyte apoptosis. Among the 34 rabbits used for modeling, 29 survived, and 25 showed punctate or regional abnormal signals on micro-CT. The success rate of establishing the early ONFH model in rabbits in this experiment was 73.53% (25/34) (Figures 7A,B).

#### 3.4.2 Treatment of CD and post-operative condition of the rabbits

All surgical operations were completed in 30 min, with an incision of 2 cm and intraoperative bleeding of 5–8 mL. A bone tunnel with a diameter of approximately 4 mm and a length of approximately 12 mm was created along the long axis of the femoral neck, and the rods of the PT group and PTB group were respectively compressed and inserted into the bone tunnel and rotated to secure them (Figure 7C). All rabbits were awake 1 h after surgery. The rabbits’ diet and behavior returned to normal within 3 days after surgery. No rabbits developed femoral fractures or hip dislocation within 4 weeks after surgery. Additionally, no infections occurred during this period.

#### 3.4.3 Histological observation of important organs of rabbits

**FIGURE 8:**
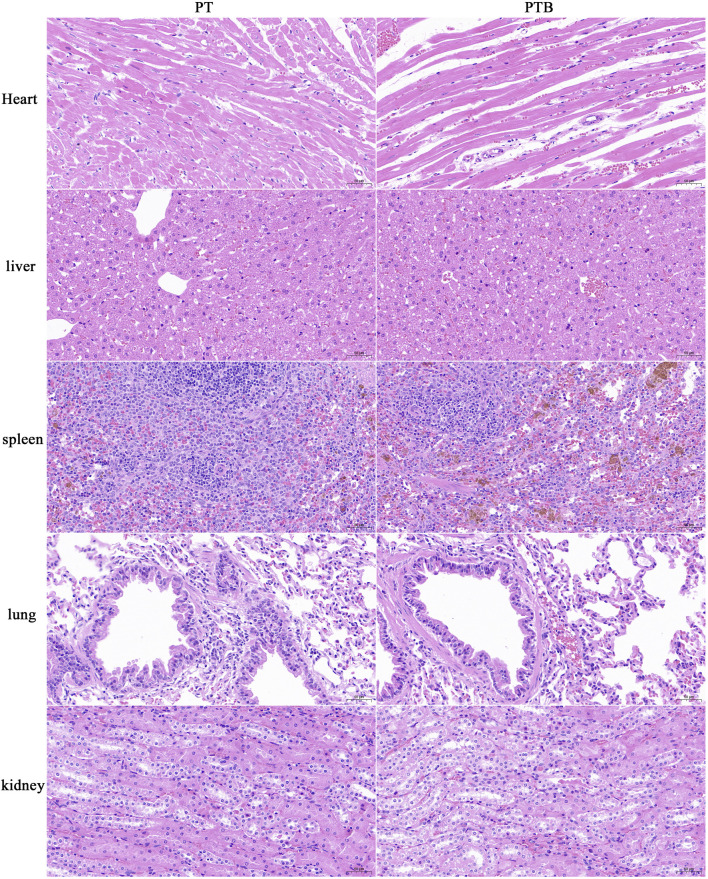
HE staining of the important organs (x20).

The heart, liver, spleen, lung and kidney were retrieved at weeks 12 after implantation, and organ structures as well as changes in cell structures were assessed by histological analysis. No pathological changes were observed in the cell structure of important organs in PT and PTB groups (Figure 8).

#### 3.4.4 Radiology analyses

**FIGURE 9:**
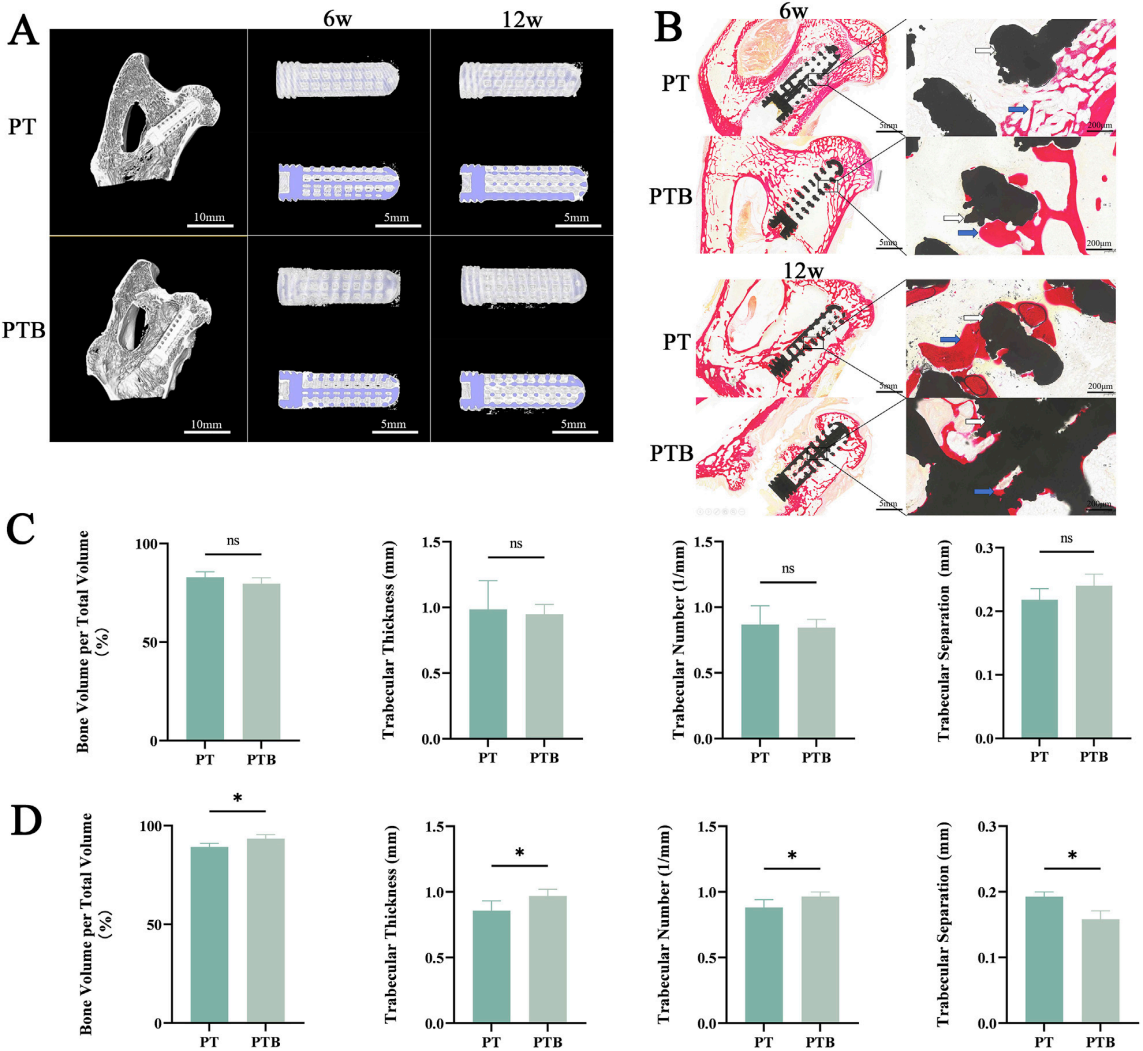
Evaluation of osteogenic effects *in vivo*. **(A)** micro-CT 3D reconstruction. Blue indicates rods. White indicates bone. **(B)** VG staining. Black indicates rods. Red indicates collagen fibers. Yellow indicates bone. **(C)** Quantification of bone volume fraction (BV/TV), trabecular thickness (Tb.Th), trabecular number (Tb.N) and trabecular separation (Tb.Sp) at week 6. Ns, p > 0.05. **(D)** Quantification of bone volume fraction (BV/TV), trabecular thickness (Tb.Th), trabecular number (Tb.N) and trabecular separation (Tb.Sp) at week 12. *, p > 0.05.

To observe condition in the early decompression tunnel and implant location, the femoral head of all rabbits was located by X-ray during surgery. All rabbits were examined by CT at 6 and 12 weeks after the operation and bone formation in the core area of the rod were observed more intuitively by micro-CT. Intraoperative and postoperative X-rays confirmed the proper positioning of the guide wire and rods (Figure 7D). Three-dimensional CT reconstructions were used to observe bone healing in the tunnel and within the rods after implantation, measuring three parameters—bone volume fraction (BV/TV), trabecular thickness (Tb.Th), trabecular number (Tb.N) and trabecular separation (Tb.Sp)—to evaluate the bone coverage in both scaffold groups. By week 6, the bone tunnels in both groups began to fill, with newly formed bone extending around the rods from the outside in. BV/TV, Tb.Th, and Tb.N were all at high levels, indicating that new bone tissue had covered and filled the tunnels and the rod bodies in both groups at week 6, with no statistically significant difference between the two groups (P > 0.05). By week 12, the BV/TV, Tb.Th, and Tb.N in the PTB group increased gradually, with the BV/TV mean nearing 100%, indicating that the newly formed bone tissue had largely filled the internal pores of the rods by this time. Compared to the PT group at the same time point, the difference was statistically significant (P < 0.05). Furthermore, both groups exhibited an increase in bone density compared to week 6, but the increase in the PTB group was more pronounced, showing a statistically significant difference compared to the PT group (P < 0.01). Although both groups showed an increase in trabecular thickness, there was no statistical difference (P < 0.05). The three-dimensional reconstructed cross-sectional images showed that at week 6, both groups had new bone formation within the bone tunnels and the pores of the rod bodies, with more growth near the edge, while some central pores remained unfilled. By week 12, the pores around and within the rods in both groups were filled with new trabecular bone, and most pores were densely filled; however, some pores near the top and center of the rods in the PT group remained unfilled, while the filling effect of newly formed bone tissue in the central pores of the PTB group was satisfactory (Figure 9).

#### 3.4.5 Histological evaluation

All specimens were collected at weeks 6 and 12 post-implantation, and histological analysis was conducted to assess collagen fibers and changes in cancellous bone repair. At week 6 post-surgery, both groups displayed red-stained collagenous tissue and newly formed mineralized bone around the rods. A small amount of collagen fibers and new bone was also visible in the edge pores. High magnification observations showed close integration between the new tissue and the materials in both groups; however, internal tissue filling the rod pores remained sparse in both groups, with no significant difference noted. At week 12 post-surgery, both groups displayed prominent red collagen fibers and pale yellow newly formed bone filling the rod’s internal pores. The newly formed tissue had filled more than half of the internal pores, and the new collagen tissue had fused into strands that traversed the internal pores of the rods, with the center region of the rods also occupied by the new tissue. High magnification observations showed that the central region in the PT group contained newly formed fibrous and mineralized tissue. However, some pores were empty, while most were filled with red, newly formed collagen tissue. In the PTB group, the peripheral areas of the rods and internal pores tightly adhered to the newly formed collagen fibers and mineralized tissue, completely covering the internal pores (Figure 9). The histological analysis results matched the trends seen in the Micro-CT analysis.

## 4 Discussion

Osteonecrosis, particularly of the femoral head, is a debilitating condition characterized by the death of bone tissue due to insufficient blood supply, leading to collapse and severe joint dysfunction. It affects a significant number of individuals annually, with peak incidence occurring among middle-aged adults (Petek et al., 2019; Wang et al., 2018). The etiology of osteonecrosis is multifactorial, often linked to risk factors such as corticosteroid use, alcohol consumption, and trauma (Zhao et al., 2015). Since conservative treatments have limited effectiveness, there is a need to explore new therapies that can promote bone regeneration and improve patient outcomes (Murab et al., 2021; Liu et al., 2022).

Currently, the primary clinical approach for femoral head-preserving treatment involves CD-based surgical methods, including CD combined with vascularized or non-vascularized bone grafting, CD with biological adjuncts (such as concentrated stem cells), and CD with tantalum rod implantation. While these methods demonstrate some therapeutic effectiveness, they have limitations, including complex vascular anastomosis, limited availability of bone and stem cells, and the high cost of tantalum rods. Previous studies have shown that using PT rods for treating early-stage ONFH yields therapeutic effects in large animal experiments. However, issues arise as the rate of bone ingrowth in the porous structure of the rods slows in the later stages of *in vivo* repair. This slowdown does not match the local bone resorption rate in ONFH, potentially leading to subchondral bone collapse in the femoral head (Wang et al., 2020a). The effectiveness of porous titanium alloys in orthopedic surgery largely relies on their ability to promote bone matrix growth and integrate with the surrounding bone (Shah et al., 2019).

Traditional surface modification methods for titanium alloys often fail to create a uniform coating, leading to limited new bone formation and minimal ingrowth after implantation (Whitaker et al., 2021; Han et al., 2023). A hydrothermal synthesis method enables the uniform coating of BaTiO_3_ particles on PT scaffolds. Subsequent arc polarization improves the piezoelectric properties of the BaTiO_3_ coating, creating a solid foundation for effective force-electric conversion in PT scaffolds (Wu et al., 2021). Our study demonstrates that modifying the surface of BaTiO_3_ particles promotes the growth and integration of the bone matrix. This enhancement improves the long-term stability of both the bone and the porous rod, leading to reduced healing time. Notably, by week 12 of *in vivo* implantation, the central area of the rod’s pores was filled with numerous collagen fibers and trabeculae. This PTB rod shows potential in improving facilitating osteogenesis repair after CD surgery for ONFH. Additionally, this material may serve as a therapeutic strategy for diseases characterized by excessive bone resorption, such as osteoporosis and osteonecrosis (Sood et al., 2023; Cai et al., 2021; Xiong et al., 2017).

This study aims to investigate the therapeutic potential of PTB scaffolds in promoting bone regeneration in osteonecrosis models. The findings indicate that BaTiO_3_ coating significantly influences cell behavior, promoting proliferation and differentiation of osteogenic cells while modulating inflammatory responses (Mao et al., 2022). These findings highlight the potential of utilizing biocompatible materials with piezoelectric properties to develop innovative treatments for osteonecrosis and other bone-related disorders (Khare et al., 2020; Tandon et al., 2018; Heng et al., 2023). Previous studies on the bioactivity of implant materials have mostly focused on static culture or simulated immersion, overlooking the complex mechanical environment and local microelectric field effects *in vivo* (Ball et al., 2014; Shokrollahi et al., 2017). In this experiment, we used a dynamic loading device to co-culture cells with materials, simulating how these materials behave in bodily fluids and microelectrical environments. This approach offers a more accurate representation of the *in vivo* conditions of implant materials. It also establishes a foundation for investigating the effects of piezoelectricity on macrophage proliferation and osteoclast formation (Tang et al., 2017). Through *in vitro* cell experiments, we observed that the piezoelectric potential generated by the PTB scaffold under simulated *in vivo* mechanical stress promotes cell proliferation and maintains a favorable condition.

Additionally, the bioelectric field generated by the BaTiO_3_ coating significantly enhances bone integration within the PT (Liu et al., 2020). In previous studies, we have demonstrated the excellent performance of PTB scaffolds in promoting angiogenesis and bone repair. Using the same method, we created interbody fusion devices and tested them in rabbit and sheep models, yielding comparable positive results in bone integration (Wu et al., 2023; Liu et al., 2020). The supporting rods evaluated in this study exhibit fully interconnected pores with adjustable pore sizes (855 ± 87 μm) and porosity (74.548% ± 0.781%). Moreover, the processes used to prepare and polarize the coating minimally affect the porous structure and mechanical properties of the rods (P < 0.05). This indicates that BaTiO_3_ particles do not significantly affect the titanium alloy’s macroscopic structure during this process. The rods can still maintain or approach the optimal range for bone growth *in vivo* in terms of pore size and porosity while meeting mechanical and strength requirements (Wang et al., 2021; Li et al., 2010; Biemond et al., 2011). Furthermore, the polarization process enhances the piezoelectric potential of the scaffolds (0.667 ± 0.158 pC/N), effectively mimicking the piezoelectricity of natural bone (0.7–2.3 pC/N) to achieve a biomimetic effect. The surface morphology shows a significant increase in the roughness of the scaffolds after coating (Ra = 813 nm), with a surface made up of uniformly distributed nanoparticles. This rough particle surface creates binding sites for early cell adhesion, which promotes cell proliferation and differentiation (Hakki et al., 2012; Wang et al., 2020b).

The findings of this study provide critical insights into the molecular mechanisms and signaling pathways involved in the osteogenic process, particularly in the context of the PTB scaffolds (Sood et al., 2023). Previous studies have shown that piezoelectric stimulation elevates intracellular Ca^2+^ concentrations and enhances p38 MAPK phosphorylation, thereby upregulating Osterix expression and promoting the osteogenic differentiation of mesenchymal stem cells (MSCs). It can also increase the secretion of Platelet-Derived Growth Factor BB (PDGF-BB) by human umbilical vein endothelial cells (HUVECs) and, via an autocrine mechanism, strengthen the PDGF-BB/PDGFR-β signaling axis and downstream effectors, thereby promoting vascularization of bone tissue (Liu et al., 2020). The change of macrophage proliferation and differentiation in the presence of BaTiO_3_ can be attributed to the unique piezoelectric properties of the material (Tandon et al., 2018; Sood et al., 2023; Mao et al., 2022). Specifically, during the bone repair process, excessive activity of osteoclasts often leads to poor bone tissue resorption and formation, ultimately resulting in repair failure and nonunion (Mediero et al., 2016). During the differentiation and maturation of osteoclasts, the RANK/RANKL/OPG system serves as a pivotal regulatory hub, acting as a key signaling pathway for the regulation of osteoclast differentiation and maturation (Srirussam et al., 2019; Boyce and Xing, 2008; Xiong et al., 2017). RANKL is considered the most important factor in promoting the differentiation and functional activity of osteoclasts, as it binds to RANK-expressing osteoclast precursors, thereby recruiting downstream tumor necrosis factor receptor-associated factors (TRAFs) to a specific domain in the cytoplasm, sequentially activating NF-κB, c-Fos, and NFATc1, which mediates the osteoclast differentiation process (Boyce and Xing, 2008; Matsuike et al., 2018). This study indicates that the BaTiO_3_ coating not only effectively reduces the expression of osteoclast markers Cathepsin K and Trap but also decreases the expression of the surface receptor RANK on osteoclast precursors, which effectively reduced the rate of osteoclast precursor cell differentiation (Chang et al., 2006). Moreover, the implications of these results extend beyond basic biology, offering promising applications in regenerative medicine. The observed increase in alkaline phosphatase activity and calcium deposition in the presence of BaTiO_3_ indicates enhanced osteogenic potential, making it a candidate for improving bone healing in clinical settings. The interplay between the material properties and cellular behavior underscores the importance of biomaterial design in tissue engineering (Zaszczyńska et al., 2020).

The immune response to biomaterials is another crucial aspect highlighted in this study. The interaction between the immune system and the biomaterial surface can significantly influence the healing process (Whitaker et al., 2021; Hu et al., 2022; Sung et al., 2023). The findings suggest that BaTiO_3_ not only promotes osteogenesis but may also reduce inflammatory responses, potentially leading to a more favorable environment for bone regeneration (Mao et al., 2022; Ball et al., 2014). In the experiment, macrophages in the PTB group exhibited a polarization-like state (either M1 or M2 type), which may contribute to the reduction in osteoclast numbers. Additionally, M1 macrophages stimulate osteoclast precursor cells to differentiate into osteoclasts by secreting pro-inflammatory cytokines (such as TNF-α, IL-6, and IL-1β), whereas bioelectric stimulation can reduce TNF-α secretion, thereby decreasing osteoclast differentiation (Srirussam et al., 2019). For macrophages, changes in morphology may indicate differences in polarization or functional states. Currently, M2 macrophages are generally considered alternatively activated anti-inflammatory macrophages, while M1 macrophages are pro-inflammatory macrophages (Wu et al., 2023; McWhorter et al., 2013). The ability of M1/M2 macrophages to further differentiate into osteoclasts remains unclear; however, some studies suggest that M1 macrophages may serve as potential precursors for osteoclasts (Yao et al., 2021). Our previous work indicates that piezoelectric stimulation can promote macrophage M2 polarization by inhibiting the MAPK cascade and by enhancing ATP synthesis and oxidative phosphorylation (Wu et al., 2023). From this perspective, the microcurrent stimulation generated by piezoelectric materials induces a functional shift in macrophages from the pro-inflammatory M1 type to the repair-promoting M2 type, which may explain the observed reduction in osteoclast numbers (Wu et al., 2023). Understanding these immune mechanisms is essential for the development of next-generation biomaterials that not only support tissue regeneration but also integrate seamlessly with the host’s immune system, ultimately improving patient outcomes in orthopedic surgeries and treatments for bone-related diseases (Tandon et al., 2018; Zheng et al., 2020).

This study acknowledges several limitations that warrant consideration. Firstly, the relatively small sample size may restrict the statistical power and generalizability of the findings. Moreover, the absence of clinical validation limits the applicability of the results to real-world scenarios. Additionally, the reliance on hormonal rabbit models without clinical trials can lead to gaps in understanding the complex interactions that occur within biological systems. These limitations underscore the necessity for further investigations with larger cohorts and clinical studies to corroborate the efficacy of the PTB scaffolds in treating osteonecrosis.

## 5 Conclusion

This study emphasizes the significant potential of BaTiO_3_-coated titanium alloy scaffolds for enhancing cellular proliferation, modulating signaling pathways, and improving biocompatibility. These characteristics suggest that such materials may play a crucial role in advancing bone tissue engineering. The insights gained from this research provide a foundation for the development of novel osteogenic materials, which could be transformative in clinical applications aimed at improving outcomes for patients with osteonecrosis. Future studies should aim to address the identified limitations while exploring the broader implications of these materials in regenerative medicine.

## Data Availability

The raw data supporting the conclusions of this article will be made available by the authors, without undue reservation.
